# Asia-Oceania HUPO: Past, Present, and Future

**DOI:** 10.1016/j.mcpro.2021.100048

**Published:** 2021-01-16

**Authors:** Yasushi Ishihama, Yu-Ju Chen, Je-Yoel Cho, Max Ching Ming Chung, Stuart J. Cordwell, Teck Yew Low, Terence Chuen Wai Poon, Ho Jeong Kwon

**Affiliations:** 1 Graduate School of Pharmaceutical Sciences, Kyoto University, Kyoto, Japan; 2 Institute of Chemistry, Academia Sinica, Taipei, Taiwan; 3 Department of Biochemistry, BK21 PLUS Program for Creative Veterinary Science Research and Research Institute for Veterinary Science, College of Veterinary Medicine, Seoul National University, Seoul, South Korea; 4 Department of Biochemistry, Yong Loo Lin School of Medicine, National University of Singapore, Singapore; 5 School of Life and Environmental Sciences and Sydney Mass Spectrometry, The University of Sydney, Sydney, Australia; 6 UKM Medical Molecular Biology Institute (UMBI), Universiti Kebangsaan Malaysia, Kuala Lumpur, Malaysia; 7 Pilot Laboratory, Proteomics Core, Institute of Translational Medicine, Centre for Precision Medicine Research and Training, Faculty of Health Sciences, University of Macau, Macau SAR, China; 8 Chemical Genomics GRL, Department of Biotechnology, Yonsei University, Seoul, South Korea

**Keywords:** proteomics organization, Asia-Oceania, HUPO, AOHUPO, proteomics community

## Abstract

The Asia-Oceania Human Proteome Organization (AOHUPO; www.aohupo.org) was officially founded on June 7, 2001, by Richard J. Simpson (Australia), Akira Tsugita (Japan), and Young-Ki Paik (Korea) and launched on October 1–4, 2001, at the second scientific meeting of the International Proteomics Conference held in Canberra, Australia. Inaugural council members of the AOHUPO elected were Richard J. Simpson (Australia, president), Qi-Chang Xia (China), Kazuyuki Nakamura (Japan), Akira Tsugita (Japan, VIce President), Young-Ki Paik (Korea, secretary general), Mike Hubbard (New Zealand), Max C. M. Chung (Singapore), Shui-Tien Chen (Taiwan), and John Bennett (Philippines). The first AOHUPO conference was held on March 26–27, 2002, at the Seoul National University, Seoul, Korea, conjointly with the second Annual Meeting of KHUPO. Since then, biennial AOHUPO conferences have been held in Taipei (2004), Singapore (2006), Cairns (2008), Hyderabad (2010), Beijing (2012), Bangkok (2014), Sun Moon Lake (2016), and Osaka (2018). The 10th AOHUPO conference is scheduled to be held in Busan on June 30 to July 2, 2021, to celebrate our 20th anniversary.

## Highlights

• AOHUPO celebrates its 20th anniversary in 2021.
• The diversity of the AOHUPO society is featured.
• The past and current activities as well as the future direction are reported.

## Graphical Abstract

**Figure:**
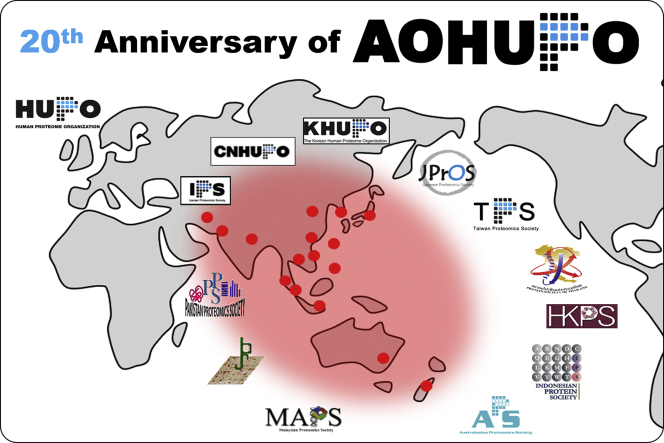

## In Brief

We summarized the activities of the AOHUPO over the past 20 years, from its foundation to the present. The AOHUPO is the only regional HUPO organization that straddles the northern and southern hemispheres, and the needs of the society for proteomics are not uniform. Despite this diversity, the research in the AOHUPO has made steady progress, and we believe that this pace of evolution can be further accelerated in the next 20 years with the addition of younger generations.

## History of the AOHUPO

**Fig. 1:**
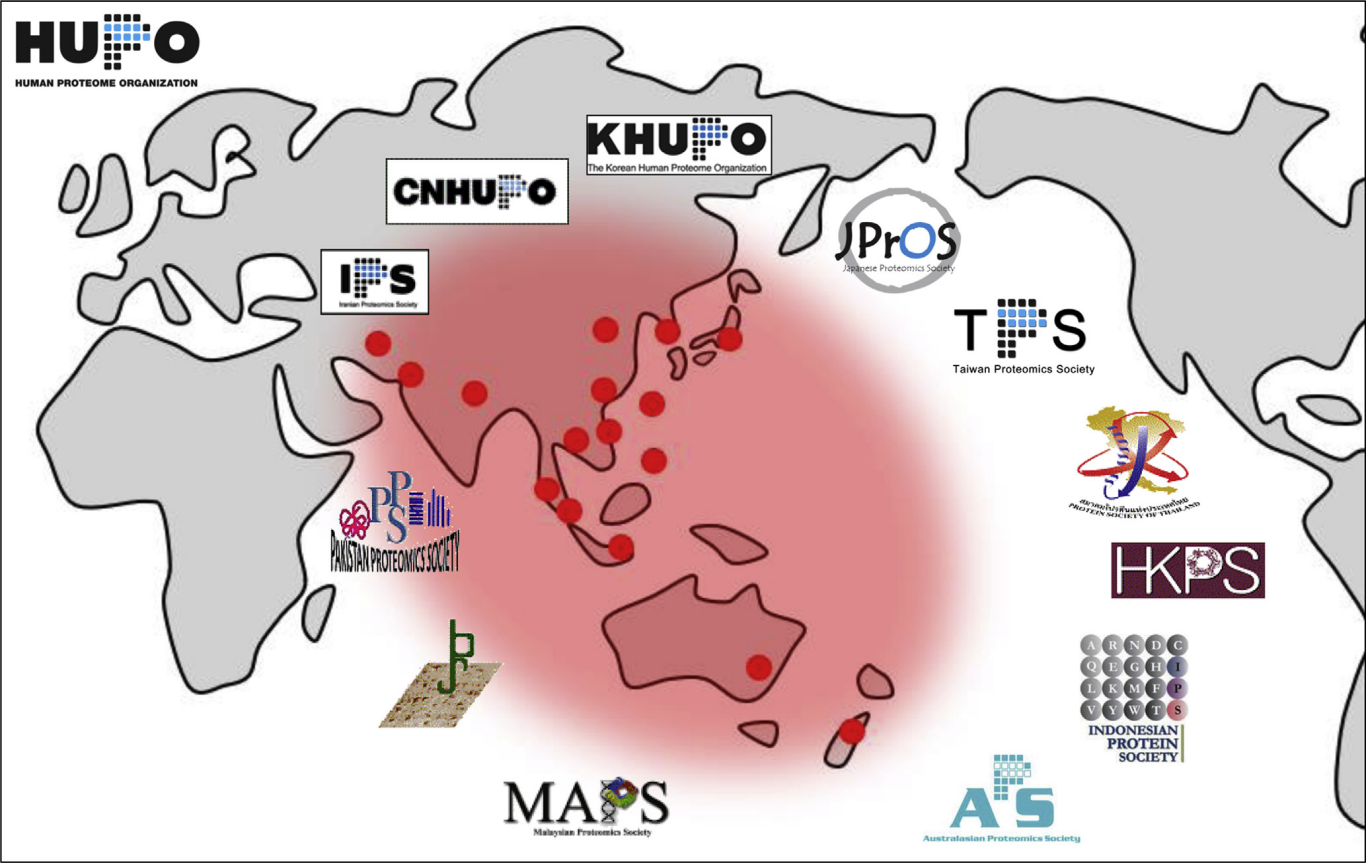
AOHUPO regional society members. AOHUPO, Asia-Oceania Human Proteome Organization.

The Asia-Oceania Human Proteome Organization (AOHUPO; www.aohupo.org) was officially founded on June 7, 2001, by Richard J. Simpson (Australia), Akira Tsugita (Japan), and Young-Ki Paik (Korea) and launched on October 1 to 4, 2001, at the second scientific meeting of the International Proteomics Conference held in Canberra, Australia. Inaugural council members of the AOHUPO elected were Richard J. Simpson (Australia, President), Qi-Chang Xia (China), Kazuyuki Nakamura (Japan), Akira Tsugita (Japan, vice president), Young-Ki Paik (Korea, secretary general), Mike Hubbard (New Zealand), Max C. M. Chung (Singapore), Shui-Tien Chen (Taiwan), and John Bennett (Philippines) (1, 2). The first AOHUPO conference was held on March 26 to 27, 2002, at the Seoul National University, Seoul, Korea, conjointly with the second Annual Meeting of KHUPO (Korean HUPO). Since then, biennial AOHUPO conferences have been held in Taipei (2004), Singapore (2006), Cairns (2008), Hyderabad (2010), Beijing (2012), Bangkok (2014), Sun Moon Lake (2016), and Osaka (2018) (supplemental Table S1). The 10th AOHUPO conference is scheduled to be held in Busan on June 30 to July 2, 2021, to celebrate our 20th anniversary (Fig. 1).

Over the past 20 years, the number of participating regions has increased from 8 (2001) to 13 (2005) and 17 (2020) regional societies. These include Australia, China, Hong Kong, India, Indonesia, Iran, Japan, Korea, Malaysia, New Zealand, Pakistan, Philippines, Singapore, Taiwan, Thailand, and Vietnam, forming Asia-Oceania alliance in addition to the international HUPO (Fig. 1). The AOHUPO has been led by Richard Simpson (Australia), Young-Ki Paik (Korea), Kazuyuki Nakamura (Japan), Fuchu He (China), and Max C. M. Chung (Singapore) as past presidents and the current president Ho Jeong Kwon (Korea) together with council members from each region.

The main aim of the AOHUPO is not only to promote and coordinate the activities of each regional proteomics community but also to facilitate the intercommunity activities through AOHUPO initiatives such as Membrane Proteome Initiative led by Bill Jordan (New Zealand) (3). Recently, attempts to unveil missing proteins, including the Membrane Proteome Initiative results (4), have resulted in reporting a 90.4% complete high-stringency human proteome blueprint through the chromosome-based human proteome project in HUPO (5). Two more initiatives such as *Chemical Proteomics* and *Nuclear Proteomics* led by two prominent scientists Ho Jeong Kwon and Jun Qin, respectively, are on-going with participation by the AOHUPO community (6). In addition, bilateral speaker exchange programs for annual conferences of each society have been established, for example, Korea-Japan, Taiwan-Korea, Australasia-Korea, Japan-Taiwan, and Korea-China, to promote networking activities for prominent or young scholars.

The AOHUPO has also served as the gate for the international HUPO because the AOHUPO covers the entire region of the “HUPO Eastern Region,” which is one of three region units in the HUPO to be responsible for the HUPO World Congress held in each region every 3 years. So far, the AOHUPO has contributed to hosting five successful HUPO Congresses in the recent past. These were in Beijing (2004), Seoul (2007), Sydney (2010), Yokohama (2013), Taipei (2016), and Adelaide (2019). In addition, to elect HUPO council members annually, the AOHUPO has helped to identify and nominate suitable diversity candidates from the HUPO eastern region to the HUPO Nomination and Election Committee for their consideration and endorsement by HUPO members. So far, the council members elected from the AO region have been actively contributing to HUPO activities as president or executive council members (supplemental Table S2). Moving forward, the AOHUPO and its council members are looking forward to playing an even more active role in HUPO affairs, and Yu-Ju Chen (Taiwan) will become the first female HUPO president from the AO region in 2021, which is the 20th anniversary year of the AOHUPO.

## Diversity in the AOHUPO

In the proteomics community, the AOHUPO is the second largest organization geographically after the international HUPO. However, unlike the HUPO, the AOHUPO does not allow personal membership, whereas the HUPO does not have to be a member of a regional academic organization to join. AOHUPO members are the representative academic organizations in each AO region. Of course, if such an organization is not yet in place, individuals represent their region in the AOHUPO. Academic organizations in each region can be (1) independent proteomics societies, (2) part of a protein society or biochemistry society, or (3) allied with mass spectrometry societies, depending on the circumstances in each region.

The AOHUPO is the only regional HUPO organization that straddles the northern and southern hemispheres and has great geographical, climatic, cultural, linguistic, and educational diversity. There is also a wide range of academic maturity in the conduct of proteomics research, and the needs of society for proteomics are not uniform. The current status of each regional society is summarized in supplemental Table S3. Despite this diversity, as indicated above, the proteomics research in the AOHUPO has made steady progress over the past 20 years, and we believe that this pace of evolution can be further accelerated in the next 20 years with the addition of younger generations.

## Future Perspectives

One of the key features the current AOHUPO focuses on is the education/training for younger generations. Although COVID-19 has disrupted our academic activities in 2020, it is also clear that in a globalized cyber society, geographic or economic limitations will not be as much of a problem. These are definite tailwinds for the AOHUPO, and we will continue to actively embrace digital transformation in our educational activities for the next 20 years. In this regards, the AOHUPO online educational series are being organized by Terence Poon and Ho Jeong Kwon as chairs together with the organizing committee of Teck Yew Low, Maxey Chung, Stuart Cordwell, Yasushi Ishihama, Yu-Ju Chen, and Je-Yoel Cho. The AOHUPO online educational series will focus on a number of ‘next-generation proteomics’–related topics presented by top caliber scientists of the field that provide new insights into future expansion of proteomics with related science and technologies for human well-being. Through conventional offline and current online mutual communications, the AOHUPO will keep on developing our mutual communications and collaborative activities in coming next 20 years.

## Conflict of interest

The authors declare no competing interests.
